# P-2269. Mixed Fungal Infection in Patients with Hematological Malignancies

**DOI:** 10.1093/ofid/ofae631.2422

**Published:** 2025-01-29

**Authors:** Isabel C Ramirez

**Affiliations:** Hospital Pablo Tobon Uribe, Universidad de Antioquia, Medellin, Antioquia, Colombia

## Abstract

**Background:**

Invasive fungal infection (IFI) is a complication of neutropenia related to hematological malignancy that carries high mortality. Invasive candidiasis remains the predominant cause of yeast infection, followed by molds, primarily *Aspergillus*, *Fusarium*, and Mucorales. The clinical spectrum varies, with fungemia and pulmonary IFI being the most common; however, sinusitis, sinopulmonary and disseminated diseases also occur or may have overlapping manifestations. Commensalism and opportunism between microorganisms can favor superinfection as has been previously demonstrated with bacteria and yeasts, virus and mold species, however, mixed fungal infections (MFI) of both yeast and molds are rarely found simultaneously.

Characteristics of patients with mixed fungal infection

**Figure:**
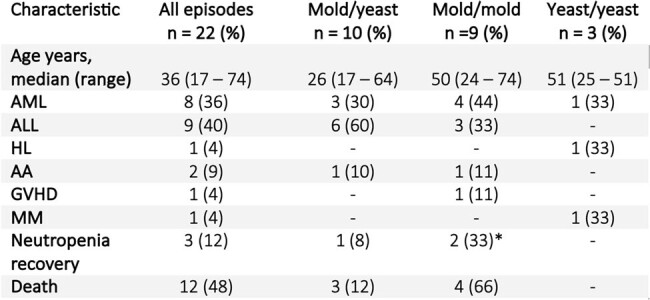

**Methods:**

Medical records and laboratory data of patients with hematological malignancies with IFI were retrospectively reviewed over a 10-year period at a tertiary care hospital. Fungal infection was diagnosed according to the EORTC/MSG criteria, and MFI were defined as the simultaneous isolation of different fungal species in the same or in different representative microbiological specimens.

**Results:**

22 episodes of MFI were found. In 90% of cases it was associated with prolonged neutropenia, most patients had acute leukemias. The most common manifestations were pulmonary IFI and fungemia (10/22), followed by sino-pulmonary IFI and fungemia. Infection by yeast and molds occurred in 13/22 cases and by different species of molds in 9/22 cases. The most common MFI was aspergillosis associated with invasive candidiasis (7/22), followed by aspergillosis and fusariosis (3/22) and aspergillosis associated with *Curvularia* infection (3/22). Breakthrough infection occurred in 11 of 22 cases. Mortality was 50%, those who survived, all resolved neutropenia.

**Conclusion:**

MFI are rare and often associated with high mortality, neutropenia resolution is an important factor for survival. Mold infection is usually manifested with sino-pulmonary involvement, so it is considered that it is caused by the same fungus. However, this study demonstrates that the etiology of sinusitis may be different from that of pulmonary involvement and, therefore, highlights the need to emphasize invasive diagnosis at each of the sites involved.

**Disclosures:**

All Authors: No reported disclosures

